# Supplementary material for: Chemical, Textural and Antioxidant Properties of Oat-Fermented Beverages with Different Starter Lactic Acid Bacteria and Pectin
Source: BioTech (Basel). 2024 Sep 25;13(4):38. doi: 10.3390/biotech13040038 (PMC11503288; doi:10.3390/biotech13040038)
Supplement: Supplementary file 1 [file biotech-13-00038-s001.zip › biotech-3157566-supplementary.pdf]

Table S1. The sensory evaluation criteria for oat beverage.

Indicator	Description	Evaluation criteria					
		0	⩽1	⩽2	⩽3	⩽4	⩽5
**Consistency**	A set of rheological characteristics of products perceived by mechanical and tactile receptors. It is reasonable to understand consistency as a characteristic of mobility (density) of viscous liquids	liquid	not thickened	not thickened	thickened	viscous, homogeneous	thick, homogeneous
Texture	A set of mechanical, geometric and surface characteristics of a product that are perceived by mechanical, tactile and, where possible, visual and auditory receptors. Texture is perceived tactilely in the oral cavity when consuming the product with the involvement of elements of mechanical impact on the product from the teeth, tongue, palate (pressing, crushing, chewing). It forms the «body» of the product.	watery	watery	watery	light, not watery	elastic, velvety	elastic, gummy, enveloping.
Colour	A characteristic of the color or color scheme of a product formed by visual evaluation of the product.	unpleasa nt	neutral	neutral	typical of this raw material	pleasant, with a beige shade	pleasant, pronounced
Taste	The presence of flavor, per se, perceived by the receptors initially upon contact with the product.	unpleasa nt	not pronounced	weak	weakly pronounced	pronounced	pleasant, pronounced
Aftertaste	A set of residual receptor responses after exposure to a product on the oral cavity, tongue, palate.	no aftertaste	insignificant	insignificant	significant	developed	long aftertaste, harmonious
Smell	The presence of an odor, per se, perceived by the receptors initially upon contact with the product.	unpleasa nt	neutral	weak	pleasant	pronounced, typical	pronounced, strong
Flavour	The totality of all the elements that form the overall perception of aroma-forming sensations by the senses of touch.	no flavour	not pronounced	weak	significant	developed	intense, balanced
General Acceptabilit y	The overall assessment of consumer properties of the product according to the totality of all indicators.	unsatisfa ctory	unsatisfactory	poor	satisfactory	good	excellent
